# Shaping nursing identity: Empathy and personality in Chinese undergraduate education

**DOI:** 10.1371/journal.pone.0361110

**Published:** 2026-10-23

**Authors:** Jie Li, Qinghua Zhao, Zhongchen Luo, Fuying Li, Xia Li, Jiao Tang

**Affiliations:** 1 Mental Health Center, West China Hospital / West China School of Nursing, Sichuan University, Chengdu, China; 2 Department of Nursing, The First Affiliated Hospital of Chongqing Medical University, Chongqing, China; 3 School of Nursing, Guizhou Medical University, Guiyang, China; 4 School of Nursing, Chongqing Medical University, Chongqing, China; 5 Intensive Care Unit, The Second Affiliated Hospital of Chongqing Medical University, Chongqing, China; Ataturk University, Faculty of Pharmacy, TÜRKIYE

## Abstract

**Background:**

Professional identity is fundamental to nursing education and influences students’ academic development, clinical practice, and career commitment. This study examined the associations of empathy and Big Five personality traits with professional identity among Chinese undergraduate nursing students.

**Methods:**

A cross-sectional observational study was conducted among undergraduate nursing students in Chongqing, China. Participants completed the Interpersonal Reactivity Index–Chinese version (IRI-C), Ten-Item Personality Inventory–Chinese version (TIPI-C), and Professional Identity Questionnaire for Medical Students (PIQMS). Data were analysed using Pearson correlation and multiple linear regression analyses.

**Results:**

A total of 274 nursing undergraduates were included. Professional identity was positively correlated with overall empathy (*r* = 0.196, *P* < 0.01) and personality traits (*r* = 0.407, *P* < 0.001). In multivariable regression analyses, more positive perceptions of nurse–patient relationships (*β* = 0.231), perspective taking (*β* = 0.219), conscientiousness (*β* = 0.204), personal distress (β = 0.154), extraversion (*β* = 0.132), and neuroticism (*β* = 0.131) were independently associated with higher professional identity. The model explained 28% of the variance in professional identity (adjusted *R*² = 0.28).

**Conclusion:**

Professional identity among nursing undergraduates was associated with empathy, personality traits, and perceptions of nurse–patient relationships. These findings support the integration of empathy-focused training and personality-informed educational strategies into undergraduate nursing education to strengthen professional identity.

## 1. Introduction

Professional identity plays a pivotal role in nursing education, as it shapes how future nurses perceive their professional responsibilities and influences their ability to deliver high-quality care and improve patient outcomes [1–4]. Professional identity refers to the self-concept based on the values, beliefs, norms, and role expectations associated with a profession [5]. Its development is dynamic and extends across the professional lifespan, with undergraduate education representing a particularly formative period [6]. A well-developed professional identity has been associated with stronger academic motivation, enhanced clinical competence, clearer career commitment, and better mental well-being [7–11], whereas a weaker identity may undermine long-term professional development and career sustainability. Although prior research has identified factors such as personal experiences [12–14], and mental health indicators [11,15,16], the role of stable individual traits remains less clearly understood.

Nursing students are not only future professionals but also individuals whose personal traits shape their learning experiences and professional development [17]. Individual traits significantly impact life performance, health outcomes, life satisfaction, formation of relationships between nurses and patients, knowledge acquisition, academic performance, and career aspirations of nursing students [18]. However, the extent to which these individual traits contribute to the formation of professional identity in nursing education remains insufficiently understood.

Empathy, a multidimensional construct encompassing cognitive and affective components, has been widely recognized as a core attribute in nursing professions. Substantial evidence among medical students indicates that higher levels of empathy are associated with better clinical competence, improved patient outcomes, and enhanced professional development [19,20]. Empathy is also considered fundamental to the development of a strong professional identity [21,22], as it enables nursing students to understand patients’ perspectives and respond appropriately to their emotions and care needs [23]. Although prior research has documented a positive association between empathy and professional identity in medical students, limited research has examined which dimensions of empathy are most relevant to professional identity among nursing undergraduates [16,24,25].

Personality refers to psychological traits that contribute to an individual’s enduring and unique patterns of emotions, thoughts, and actions [26]. The Five-Factor Model (FFM), commonly referred to as the Big Five, is the most extensively validated framework for describing personality traits, including agreeableness, conscientiousness, extraversion, neuroticism, and openness [17,27,28]. These traits have been shown to influence academic achievement, interpersonal competence, and professional performance among healthcare students [18]. While some studies have explored associations between personality traits and professional identity in various disciplines [29,30], research specifically examining how the Big Five traits relate to professional identity in nursing undergraduates remains limited and fragmented [31].

Although empathy and personality traits are each related to professional development, their combined associations with professional identity in nursing undergraduates remain insufficiently understood. Understanding how these individual differences relate to identity formation may clarify the psychological foundations of professional development in nursing education. Therefore, this study examines the associations of empathy and Big Five personality traits with professional identity among Chinese nursing undergraduates.

## 2. Methods

### 2.1 Study design

This cross-sectional observational study was conducted was conducted between 1 March and 18 July 2024 to explore the professional identity of nursing students in a medical school that provides comprehensive undergraduate education. This study was reported in accordance with the STROBE guidelines for cross-sectional studies.

### 2.2 Participants

The study focused on a cohort of dedicated full-time undergraduate nursing students. The participants were enrolled via a nonprobability sampling technique known as convenience sampling. The inclusion criteria for this study required participants were full-time undergraduate nursing students who had completed at least one semester of study, and provided electronic informed consent before accessing the questionnaire. The sole exclusion criterion was a current leave of absence.

An a priori sample size calculation was performed using G*Power version 3.1.9.7 (Heinrich Heine University Düsseldorf, Düsseldorf, Germany). The calculation was based on linear multiple regression using the fixed model, (*R^2^*) deviation from zero procedure. Assuming a medium effect size of Cohen’s (f^2^ = 0.15), a two-sided significance level of α = 0.05, statistical power of 0.95, and 9 predictors, the minimum required sample size was 166 participants. The final analytic sample of 274 participants exceeded this requirement.

### 2.3 Procedure

The online questionnaire was developed and deployed using Questionnaire Star, a reputable online survey platform developed by Changsha Ranxing Information Technology Co., Ltd. The first page of the online questionnaire contained an informed consent form detailing participants’ rights, including the freedom to participate or withdraw from the survey at any time without repercussions. The second page of the online questionnaire specified the study’s inclusion and exclusion criteria to verify participant eligibility. Eligible participants were directed to the main survey interface, while those who did not meet the criteria were guided to exit the link. The entire survey took approximately 5 minutes to complete.

The online questionnaire was distributed via WeChat, the most widely used mobile instant messaging platform in China. Academic advisors from the School of Nursing were invited to distribute the survey link through existing WeChat groups established for undergraduate nursing students across different academic years. Each WeChat group comprised approximately 100–160 students, with 14 groups included in total. Meanwhile, the questionnaire could be forwarded across multiple student communication groups, enabling broad dissemination across student networks. However, forwarding between groups could not be tracked. Consequently, the exact number of students who received or viewed the survey invitation could not be determined and a formal study response rate could not be calculated.

### 2.4 Measures

The e-questionnaire utilized in this study encompassed social-demographic information such as age, gender, academic year, student cadre position, involvement as a student volunteer, and perspectives of the nurse–patient relationship.

*Empathy:* Nursing students’ empathy was assessed using the Interpersonal Reactivity Index–C (IRI-C), originally developed by Davis [32], translated into Chinese by Zheng for using among medical students, including nursing students in China [33]. The IRI-C questionnaire comprises 22 items, categorized into perspective taking, fantasy, empathic concern, and personal distress, with each dimension consisting of 5–6 items [34]. Perspective taking assesses cognitive empathy, empathic concern measures emotional empathy, fantasy evaluates engagement with emotions in fictional works, and personal distress examines self-involvement in empathy [32]. Responses are rated on a 5-point Likert scale (0–4), with a total score ranging from 0 to 88, where higher scores indicate stronger empathic abilities. The IRI-C has demonstrated good reliability and validity among Chinese undergraduate nursing students, with an overall Cronbach’s α of 0.870 [35]. In the present study, the overall Cronbach’s α coefficient was 0.755. Cronbach’s α coefficients for the four subscales were 0.794 for perspective taking, 0.845 for fantasy, 0.718 for empathic concern, and 0.678 for personal distress.

*Personality:* Personality traits were assessed using the Chinese version of the Ten-Item Personality Inventory (TIPI-C), originally developed by Gosling et al. [36], and translated and culturally adapted into Chinese by Li [37]. The TIPI-C questionnaire consists of 10 items, with 2 items each for agreeableness, conscientiousness, extraversion, neuroticism, and openness factors. Each item is rated on a 7-point Likert scale (1–7), where 1 represents “Strongly Disagree,” and 7 represents “Strongly Agree.” Items 2, 4, 6, 8, and 10 are reverse-scored. The TIPI-C has demonstrated good psychometric properties and has been validated among Chinese undergraduate nursing students, with Cronbach’s α coefficients ranging from 0.90 to 0.94 across the five personality dimensions [38]. In the present study, internal consistency of the overall instrument was acceptable (Cronbach’s α = 0.780), and split-half reliability assessed using the Spearman–Brown coefficient was 0.741, indicating satisfactory reliability in our sample.

Professional identity: Professional identity was assessed using the Professional Identity Questionnaire for Medical Students (PIQMS) to understand the professional identity of nursing students, which was initially developed by Zhang [39]. The PIQMS questionnaire is widely used among medical students, including nursing students in China [39]. The PIQMS questionnaire consists of a total of 38 items, encompassing six major dimensions: Vocational Cognition, Vocational Emotion, Vocational Commitment, Vocational Behavior, Vocational Expectation, and Vocational Values. Each item is rated on a 5-point Likert scale (1–5), where 1 represents “Strongly Disagree,” and 5 represents “Strongly Agree.” In our study, the overall Cronbach’s α coefficient for the PIQMS questionnaire was 0.950. The Cronbach’s α coefficients for the individual dimensions were as follows: 0.714 for vocational cognition, 0.909 for vocational emotion, 0.694 for vocational commitment, 0.860 for vocational behavior, 0.934 for vocational expectation, and 0.814 for vocational values.

### 2.5 Data analysis

Data were analyzed using the Statistical Package for the Social Sciences (SPSS) version 25.0 (IBM Corporation, Armonk, NY, USA). We employed Harman’s single-factor test to assess common method bias. Descriptive statistics were used to summarize participants’ characteristics and scale scores (IRI-C, TIPI-C, and PIQMS). Subsequently, a two-stage variable selection procedure was implemented, comprising univariate analysis followed by multivariable regression modeling, which is a widely accepted strategy in exploratory observational research within nursing, public health, and social sciences.

Univariate analysis serves as a systematic first-pass filter to identify variables with marginal statistical associations with the outcome. The univariate analysis methods used in this study were as follows: Independent-sample t-tests and one-way analysis of variance (ANOVA) were used to examine group differences in PIQMS scores, while Pearson correlation analyses were conducted to assess the relationships between PIQMS scores and continuous variables. In addition, a correlation heatmap was generated to visually summarize the relationships among empathy dimensions, personality traits, and professional identity. The P < 0.05 threshold used to select variables for the multivariable model is the most widely adopted cutoff for this screening approach in applied research, as it balances the retention of potentially meaningful predictors with the parsimony of the final model. Accordingly, variables with *P* < 0.05 in the univariate analysis were included in a stepwise multiple linear regression model (entry criterion *P* ≤ 0.05, removal criterion *P* ≥ 0.10) to determine factors independently associated with PIQMS. The ordinal variable “perspectives on nurse–patient relationships” (Very tension, Tension, Uncertain, Harmony, Very harmony) was treated as an ordered categorical variable and coded using numeric scores (1–5) for regression analysis, while all other continuous predictors were included as measured. This approach allowed estimation of standardized regression coefficients (*β*) to assess the relative contribution of each predictor.

Prior to interpretation, regression assumptions were examined. Normality of residuals was assessed using standardized residual plots and histograms. Influential observations were assessed using Cook’s distance, with values greater than 1 considered indicative of potentially influential cases [40]. Multicollinearity was assessed using variance inflation factors (VIFs), with values less than 10 considered acceptable [41]. To examine the robustness of the regression results, a sensitivity analysis was conducted by including demographic covariates (age, gender, academic year, student cadre, and volunteer status) in the linear regression model using the enter method. All analyses were conducted using a two-tailed significance threshold of 0.05.

### 2.6 Ethical approval

Ethical approval was obtained from the ethics committee of Chongqing Medical University (Approval No. 2022106) in accordance with the Guideline for the Administration and Ethical Review of Ethics Committees in Biomedical Research of China and the ethical principles outlined in the Declaration of Helsinki, as revised in 2024. All participants provided informed consent before completing the online questionnaire. Responses were collected anonymously, and confidentiality was maintained throughout the research process.

## 3. Results

### 3.1 Participant characteristics

We received responses from 303 questionnaires, and ultimately obtained 274 valid questionnaires with signed consent. The valid rate for the questionnaire was 90.4% (274/303). As the survey link could also be shared across multiple student communication groups and forwarding between groups could not be tracked, the exact number of students who received or viewed the invitation was unknown. Therefore, a response rate could not be calculated. The Harman’s single-factor test indicated that the first factor accounted for 23.58% of the variance, suggesting that common method bias is unlikely to be a major concern.

Among them, 62 (22.6%) were male students, and 212 (77.4%) were female students. Most of them have the student cadre position (192/274, 70.1%) and volunteer experience (244/274, 89.1%). In addition, Only 9.1% of the students perceived the relationship between nurses and patients as harmonious. The basic characteristics of the students are presented in Table 1.

Table 2 presents the scores for each dimension of the IRI-C, TIPI-C, and PIQMS questionnaires for 274 participating students. The IRI-C, TIPI-C, PIQMS means and standard deviations (SD) were 66.60 (10.04), 42.42 (4.91), and 128.23 (22.00).

**Table 1:** Sociodemographic characteristics of the study participants.

Characteristic	Mean±SD	N	%
**Age (year)**	19.87 ± 1.45		
**Gender**			
Male		62	22.6
Female		212	77.4
**Academic year**			
1st year		55	20.1
2nd year		94	34.3
3rd year		93	33.9
4th year		32	11.7
**Student cadre**			
No		82	29.9
Yes		192	70.1
Class cadre		120	62.5
Student association cadre		85	44.3
**Student volunteer**			
No		30	10.9
Yes		244	89.1
**Perspectives on nurse–patient relationships**			
Very tension		20	7.3
Tension		137	50.0
Uncertain		92	33.6
Harmony		22	8.0
Very harmony		3	1.1

**Table 2:** Descriptive statistics and internal consistency of the IRI-C, TIPI-C, and PIQMS.

Variable		Minimum of actual score	Maximum of actual score	Mean scores (Mean ±SD)	Cronbach’s alpha
**IRI-C**	Total score	37.00	94.00	66.60 ± 10.04	0.755
	Perspective taking	5.00	25.00	15.30 ± 3.73	
	Fantasy	5.00	25.00	13.11 ± 4.37	
	Empathic concern	9.00	30.00	19.22 ± 3.52	
	Personal distress	14.00	30.00	21.03 ± 3.20	
**TIPI-C**	Total score	26.00	67.00	42.42 ± 4.91	0.780
	Extraversion	3.00	14.00	8.08 ± 1.85	
	Agreeableness	4.00	14.00	8.96 ± 1.54	
	Conscientiousness	2.00	14.00	8.45 ± 1.43	
	Neuroticism	4.00	14.00	8.42 ± 1.58	
	Openness	5.00	14.00	8.51 ± 1.33	
**PIQMS**	Total score	53.00	182.00	128.23 ± 22.00	0.950
	Vocational cognition	7.00	32.00	19.91 ± 3.61	
	Vocational emotion	6.00	30.00	20.58 ± 5.01	
	Vocational commitment	4.00	20.00	13.68 ± 2.93	
	Vocational behavior	4.00	20.00	14.43 ± 3.03	
	Vocational expectation	9.00	45.00	31.08 ± 6.84	
	Vocational values	12.00	40.00	28.54 ± 4.73	

*Notes:* Abbreviations: IRI-C, Interpersonal Reactivity Index–Chinese; TIPI-C, Ten-Item Personality Inventory in China; PIQMS, Professional Identity Questionnaire for Medical Students.

### 3.2 Relationships between PIQMS and participant characteristics

Table 3 provides a comprehensive comparison of PIQMS total scores among different demographic characteristics, IRI-C, and TIPI-C. Our analysis of students’ basic characteristics revealed a significant positive correlation between perspectives on nurse–patient relationships and PIQMS total score (*F* = 18.956, *P* = 0.001), while a significant negative association was observed between age and PIQMS total score (*r* = −0.194, *P* = 0.001). Furthermore, we found a significant association between the IRI-C total score and high-PIQMS (*r* = 0.196, *P* = 0.001). However, no significant association was found between the fantasy score of IRI-C and High-PIQMS (*P* = 0.655). Nevertheless, all other dimensions in IRI-C showed a positive correlation with the PIQMS total score (*P* < 0.05). Additionally, our results revealed a significant positive correlation between the TIPI-C total score and scores for 5 dimensions with the PIQMS total score (*P* < 0.05). A correlation heatmap (Fig 1) was generated to visually summarize relationships among empathy dimensions, personality traits, and professional identity.

**Table 3:** Associations between participant characteristics, IRI-C, TIPI-C, and PIQMS.

Characteristic	PIQMS total score		
	Mean ± SD	*t/F/r*	*P* (two-tailed)
**Age (year)**		−0.194	0.001
**Gender**		−1.142	0.256
Male	125.34 ± 22.88		
Female	129.07 ± 21.72		
**Academic year**		7.657	0.054
1st year	132.49 ± 23.68		
2nd year	126.89 ± 21.87		
3rd year	126.33 ± 16.28		
4th year	122.84 ± 24.56		
**Student cadre**		−0.846	0.399
No	126.56 ± 20.74		
Yes	128.94 ± 22.53		
**Student volunteer**		−1.042	0.305
No	123.57 ± 26.47		
Yes	128.80 ± 21.38		
**Perspectives on nurse–patient relationships**		18.956	0.001
Very tension	116.80 ± 34.05		
Tension	126.35 ± 20.19		
Uncertain	128.58 ± 18.05		
Harmony	145.36 ± 23.88		
Very harmony	153.67 ± 27.65		
**IRI-C**			
Total score		0.196	0.001
Perspective taking		0.305	< 0.001
Fantasy		−0.027	0.655
Empathic concern		0.144	0.017
Personal distress		0.213	< 0.001
**TIPI-C**			
Total score		0.407	< 0.001
Extraversion		0.216	< 0.001
Agreeableness		0.259	< 0.001
Conscientiousness		0.325	< 0.001
Neuroticism		0.310	< 0.001
Openness		0.184	0.002

*Notes:* Abbreviations: IRI-C, Interpersonal Reactivity Index–Chinese; TIPI-C, Ten-Item Personality Inventory in China; PIQMS, Professional Identity Questionnaire for Medical Students.

**Fig 1:**
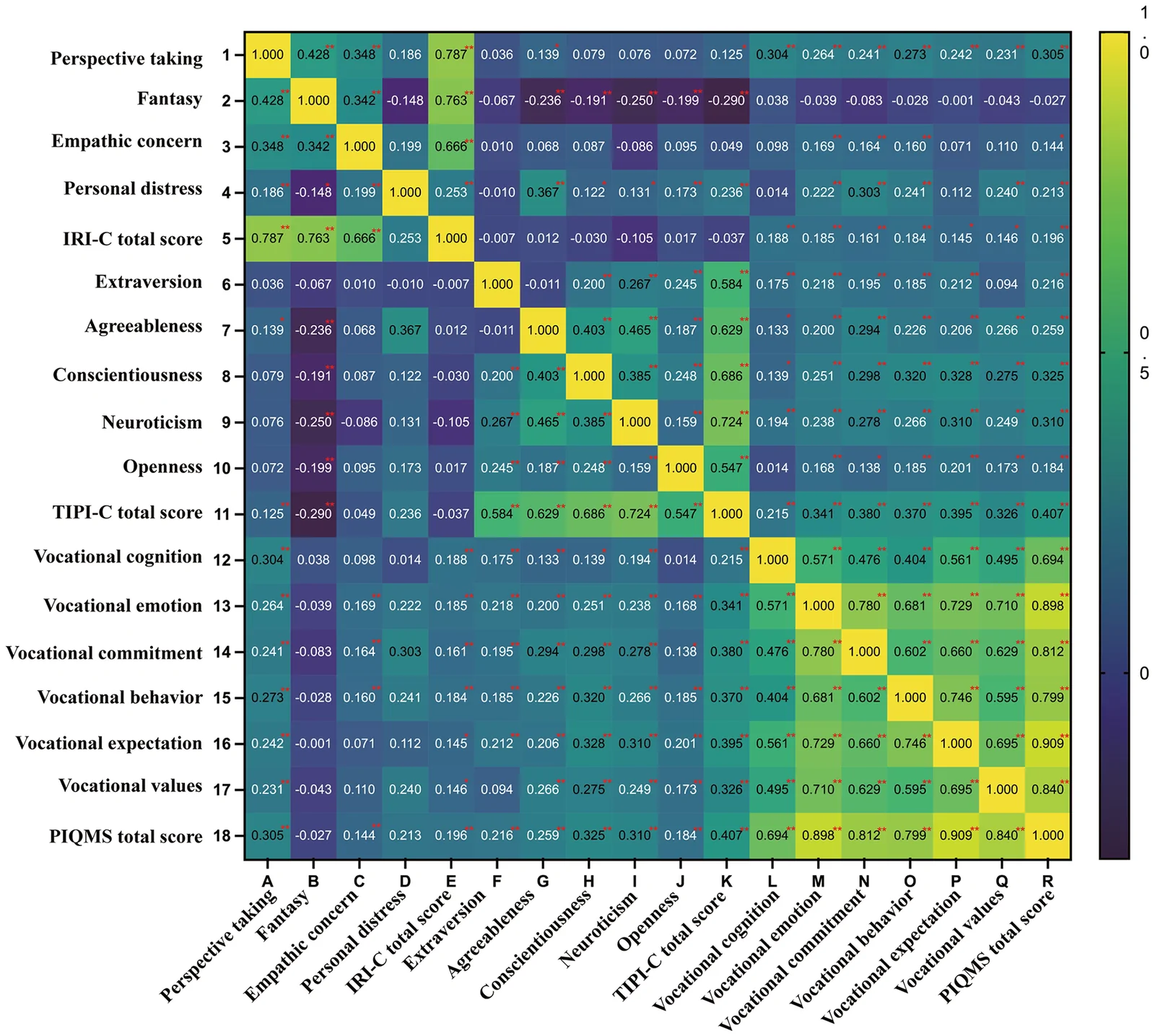
Correlation heatmap of IRI-C, TIPI-C, and PIQMS. *Notes:* Cells display Pearson’s correlation coefficients (r). Warmer colors indicate stronger positive correlations, and cooler colors indicate stronger negative correlations. *P < 0.05; **P < 0.01. Abbreviations: IRI-C, Interpersonal Reactivity Index–Chinese; TIPI-C, Ten-Item Personality Inventory in China; PIQMS, Professional Identity Questionnaire for Medical Students.

The results of the multiple linear model (Table 4) indicated that PIQMS scores were influenced by several factors. Specifically, the perspectives of the nurse–patient relationship, along with two dimensions from IRI-C (perspective taking and personal distress), and three dimensions from TIPI-C (Extraversion, conscientiousness, and neuroticism) were found to be significant predictors of PIQMS scores. Among the variables included in the model, perspectives on nurse–patient relationships had the strongest impact on PIQMS scores (*β* = 0.231), followed by perspective taking (*β* = 0.219) and conscientiousness (*β* = 0.204). The variables collectively explained 28.0% of the variance in professional identity (adjusted *R²* = 0.28, *F* = 18.667, *P* < 0.001) (Table 4). Examination of regression assumptions indicated no serious violations. Standardized residuals were within ± 3, Cook’s distance values were below 1, and all VIF values were below 10, indicating no influential cases or multicollinearity concerns.

**Table 4:** Multiple linear regression analysis of factors associated with PIQMS.

Variable	B	SE -B	*β*	*t*	*P* (two-tailed)	95% CI		VIF
						Low	Up	
Constant	15.910	11.459	/	1.388	0.166	−6.651	38.471	/
Conscientiousness	3.120	0.864	0.204	3.613	< 0.001	1.420	4.821	1.198
Perspective taking	1.292	0.313	0.219	4.134	< 0.001	0.677	1.908	1.059
Perspectives on nurse–patient relationships	6.448	1.470	0.231	4.386	< 0.001	3.554	9.342	1.048
Neuroticism	1.829	0.805	0.131	2.271	0.024	0.244	3.415	1.262
Personal distress	1.059	0.368	0.154	2.875	0.004	0.334	1.784	1.081
Extraversion	1.571	0.641	0.132	2.451	0.015	0.309	2.833	1.095

*Notes:* Model statistics: *R²* = 0.30, Adjusted *R²* = 0.28, *F* (7,266) = 18.667, *P* < 0.001. Abbreviations: B, unstandardized coefficient beta; SE-B, standard error of B; *β*, standardized coefficient beta; CI, Confidence Interval; VIF, variance inflation factor.

A sensitivity analysis was conducted by including demographic covariates (age, gender, academic year, student cadre, and volunteer status) using the enter method (Table 5). The main findings remained consistent, with perspectives on nurse–patient relationships, perspective taking, personal distress, conscientiousness, extraversion, and neuroticism remaining significant predictors. Demographic covariates were not significant (all *P* > 0.05). The model explained 27.0% of the variance in professional identity (adjusted *R²* = 0.27, *F* = 10.191, *P* < 0.001), suggesting that the primary regression results are robust to the inclusion of demographic variables.

**Table 5:** Sensitivity analysis of factors associated with PIQMS after adjustment for demographic covariates.

Variable	B	SE -B	*β*	*t*	*P* (two-tailed)	95% CI		VIF
						Low	Up	
(Constant)	47.089	32.391	/	1.454	0.147	−16.692	110.87	/
Conscientiousness	2.983	0.884	0.195	3.373	< 0.001	1.242	4.724	1.241
Perspective taking	1.244	0.317	0.211	3.923	< 0.001	0.620	1.869	1.077
Perspectives on nurse–patient relationships	6.293	1.493	0.226	4.216	< 0.001	3.354	9.232	1.068
Neuroticism	1.777	0.815	0.128	2.181	0.030	0.173	3.382	1.277
Personal distress	1.007	0.375	0.146	2.687	0.008	0.269	1.746	1.108
Extraversion	1.544	0.651	0.130	2.372	0.018	0.262	2.825	1.116
Gender	0.232	2.800	0.004	0.083	0.934	−5.282	5.745	1.062
Age	−1.435	1.361	−0.095	−1.055	0.293	−4.115	1.244	2.996
Academic year	0.688	1.958	0.032	0.351	0.726	−3.167	4.543	3.019
Student cadre	0.940	2.590	0.020	0.363	0.717	−4.160	6.039	1.087
Student volunteer	−1.159	13.651	−0.004	−0.085	0.932	−28.040	25.722	1.045

*Notes:* Model statistics: *R²* = 0.30, Adjusted *R²* = 0.27, F (10, 263) = 10.191, *P* < 0.001. Abbreviations: B, unstandardized coefficient beta; SE-B, standard error of B; *β*, standardized coefficient beta; CI, Confidence Interval; VIF, variance inflation factor.

## 4. Discussion

This study examined the associations among personality, empathy, and professional identity among undergraduate nursing students. Empathy—especially perspective-taking and personal distress—was positively associated with professional identity, while traits such as extraversion, conscientiousness, and neuroticism were also significantly associated with professional identity. Although both age and views on nurse–patient relationships were associated with students’ professional identity, only a more harmonious nurse–patient relationship remained significantly associated with professional identity in the regression analysis. Regression analysis indicated that age did not have a significant effect. These findings highlight the importance of targeted empathy training, particularly in perspective-taking and emotional empathy. Furthermore, an understanding of nursing students’ personality traits is essential for designing training tailored to individual learning styles and for supporting the development of personality traits that are associated with a stronger professional identity. Emphasising the enhancement of nurse–patient relationships, particularly by fostering harmony in interactions, may be relevant to professional identity of undergraduate nursing students.

Previous studies have already reported associations between empathy abilities and professional identity in nursing students [42,43], and our findings are consistent with previous studies and further emphasize the importance of fostering empathy in nursing education to improve students’ professional identity. Specifically, we found that perspective taking and personal distress in nursing undergraduate students were associated with professional identity. Perspective taking measures cognitive empathy, understanding and sharing others’ perspectives, while personal distress reflects a self-oriented emotional response to others’ distress, representing emotional empathy. Previous studies have suggested that prior similar experiences are associated with perspective taking [44]. Previous research suggests that perspective taking is positively related to motivation and the Big Five personality traits (except neuroticism) in adults and has also been linked to teachers’ experience and positive expectations in educational settings [45]. Educational interventions have shown potential in maintaining and enhancing empathy among medical students [46]. These findings suggest that educational strategies aimed at strengthening empathy, particularly perspective-taking skills, may support professional identity development among nursing undergraduates. Given the relatively lower reliability of the personal distress subscale, the observed association involving this dimension requires further confirmation in future studies.

This study supports the connection between personality traits and professional identity among nursing undergraduate students [30,31,47]. It underscores that personality traits are related to students’ identification with their profession. It should be noted that the TIPI-C is a very brief instrument. While it allows for efficient assessment of the Big Five traits, some dimensions may exhibit limited internal consistency, which could introduce measurement error and attenuate observed effect sizes. Our findings align with previous research, revealing a positive correlation between conscientiousness, extraversion, and openness traits and professional identity. However, regarding agreeableness, our results differ from Wang et al.’s 2023 survey [31] among business school students but agree with Wu et al.’s 2022 study [47] among nursing students, suggesting a positive relationship between agreeableness and professional identity. This discrepancy may be due to differences in professions and education levels. A meta-analysis indicates a positive link between agreeableness and organizational commitment [48], which is itself positively correlated with professional identity [49], This suggests a possible association between agreeableness and professional identity, but further research is required to confirm this connection.

Regarding neuroticism, our study diverges from previous research results [31,47,50,51], showing a modest positive association with professional identity (β = 0.131). This association remained stable after adjustment for demographic covariates in the sensitivity analysis. Traditionally, neuroticism has been associated with poorer academic performance, greater psychological distress, and less adaptive career development [52–55]. Therefore, the present finding should be interpreted cautiously. One possible explanation is that students with higher neuroticism may be more sensitive to professional expectations and role responsibilities, resulting in stronger self-reported professional identity. Such heightened vigilance may also reflect a stronger sense of professional responsibility rather than greater psychological adjustment. Alternatively, the observed association may reflect contextual influences specific to Chinese nursing education, rather than a stable psychological pattern. Residual confounding and statistical suppression cannot be excluded, particularly because psychological factors such as anxiety, resilience, and coping strategies were not measured. Furthermore, all variables were self-reported, which may have introduced response bias. Given the cross-sectional design, no causal or temporal inferences can be drawn. Future longitudinal studies incorporating broader psychological variables are needed to determine whether this association reflects a genuine psychological mechanism or a context-dependent phenomenon. Taken together, these findings suggest that personality characteristics should be considered when designing educational strategies to support professional identity development among nursing students.

Previous research findings indicated a negative correlation between the duration of medical education and professional identity. Our study results indicate that increasing age was associated with lower professional identity, independent of academic year. Given the cross-sectional design, the directionality of this association cannot be determined. The finding that 9.1% of nursing undergraduate students perceived the nurse–patient relationship as harmonious reflects relatively negative perceptions of the future working environment. Previous research suggests that perceived patient relationships are associated with professional identity [56]. This phenomenon aligns with previous research. For instance, a survey conducted by Liu et al. (2021) among medical students in Heilongjiang Province, China, found that only 6.3% of students considered the relationship between nurses and patients to be harmonious [57]. This phenomenon may be related to the frequent occurrence of doctor–patient disputes and even violent incidents in the Chinese healthcare environment. According to a survey conducted in 2015, among 1,656 doctors across various regions in China, 92.8% reported experiencing verbal abuse, 88.1% had received threats, and 81.0% had faced physical attacks [58]. However, the relationship between nurses and patients is not solely determined by individual healthcare professionals; within this binary relationship, the healthcare system, social environment, and national governance also play crucial roles. In the backdrop of this intricate interplay, the current healthcare landscape in China necessitates the combined efforts of both society and the government. Among the demographic characteristics, the perception of nursing undergraduate students regarding a more harmonious relationship between nurses and patients is the only factor independently associated with stronger professional identity, and it stands out as the most significant predictor in the results of our study. Besides providing comprehensive knowledge and advanced skills, we can further bolster training in nurse–patient communication for nursing undergraduate students [59,60]. This will contribute to fostering a more harmonious relationship between nurses and patients, complementing the development of their professional identity.

### 4.1 Limitations

This study has several limitations. First, its cross-sectional design precludes conclusions about causality or temporal sequencing; associations should be interpreted as correlational. Second, participants were recruited from a single institution using convenience sampling through WeChat groups. As participation was voluntary, the sample may not be fully representative of all undergraduate nursing students, and selection bias cannot be excluded. In addition, because the questionnaire was distributed through multiple WeChat groups, the exact number of students who received the invitation was unknown, precluding calculation of a response rate. Therefore, caution is warranted when generalizing these findings to nursing students from other institutions or educational settings. Although no significant PIQMS differences were observed across demographic subgroups, unmeasured selection effects cannot be ruled out, which may limit the generalizability of the findings. Third, all variables were self-reported, and the modest explained variance in professional identity (*R*² = 0.28) indicates that additional factors may influence outcomes. Finally, aspects such as clinical exposure quality, academic performance, and family background were not examined. Despite these limitations, the study utilized validated measurement scales, applied appropriate statistical analyses, and controlled for major demographic variables, thereby providing a methodologically sound and internally consistent examination of factors associated with professional identity among nursing students.

### 4.2 Implications and future research

The present findings suggest that professional identity formation in nursing education may be related to the combined influence of relatively stable personality traits and modifiable empathy capacities. From a practical perspective, nursing educators may consider integrating structured perspective-taking workshops, reflective patient-narrative exercises, and communication simulations to support the development of cognitive and emotional empathy [61,62]. At the same time, mentoring strategies that acknowledge individual personality differences, may help align personal dispositions with professional role expectations. Theoretically, these findings support a view of professional identity as emerging from the interaction between dispositional traits and social–educational experiences, with empathy potentially serving as a psychological pathway linking personality characteristics to professional self-concept. Future longitudinal and intervention-based studies are needed to clarify temporal relationships among variables and to examine whether empathy-focused or personality-informed educational strategies are associated with changes in professional identity over time. Further research should also explore potential mediating or moderating mechanisms and validate these associations across different cultural and institutional contexts.

## 5. Conclusion

This study identified significant associations among empathy, personality traits, and professional identity in undergraduate nursing students. Specifically, more positive perceptions of nurse–patient relationships, greater perspective taking and personal distress, and higher levels of conscientiousness, extraversion, and neuroticism were independently associated with stronger professional identity. These findings highlight the potential importance of empathy and personality in professional identity development and may inform educational strategies that foster empathy while acknowledging individual personality differences. Given the observational nature of this study, future longitudinal and intervention studies are needed to confirm these associations and clarify their temporal relationships.

## Supporting information

S1 File STROBE Statement of this study. (DOCX)
