# Supplementary material for: Shaping nursing identity: Empathy and personality in Chinese undergraduate education
Source: PLoS One. 2026 Oct 23;21(10):e0361110. doi: 10.1371/journal.pone.0361110 (PMC13693926; doi:10.1371/journal.pone.0361110)
Supplement: Supplementary file 1 [file pone.0361110.s001.docx]

STROBE Statement—Checklist of items that should be included in reports of ***cross-sectional studies***

|  | Item No | Recommendation | Page No |
| --- | --- | --- | --- |
| **Title and abstract** | 1 | (*a*) Indicate the study’s design with a commonly used term in the title or the abstract | P1-3 |
|  |  | (*b*) Provide in the abstract an informative and balanced summary of what was done and what was found | P2-3 |
| Introduction | | | |
| Background/rationale | 2 | Explain the scientific background and rationale for the investigation being reported | P4 line 43- P5 line 82 |
| Objectives | 3 | State specific objectives, including any prespecified hypotheses | P6 line 83-88 |
| Methods | | | |
| Study design | 4 | Present key elements of study design early in the paper | P6 line 90-94 |
| Setting | 5 | Describe the setting, locations, and relevant dates, including periods of recruitment, exposure, follow-up, and data collection | P7 line 108-P8 line 127 |
| Participants | 6 | (*a*) Give the eligibility criteria, and the sources and methods of selection of participants | P6 line 95-P7 line 107 |
| Variables | 7 | Clearly define all outcomes, exposures, predictors, potential confounders, and effect modifiers. Give diagnostic criteria, if applicable | P8 line 128-P10 line 169 |
| Data sources/ measurement | 8* | For each variable of interest, give sources of data and details of methods of assessment (measurement). Describe comparability of assessment methods if there is more than one group | P8 line 128-P10 line 169 |
| Bias | 9 | Describe any efforts to address potential sources of bias | P10 line170-P11 line 204 |
| Study size | 10 | Explain how the study size was arrived at | P7 lines 102-107 |
| Quantitative variables | 11 | Explain how quantitative variables were handled in the analyses. If applicable, describe which groupings were chosen and why | P10 line170-P11 line 204 |
| Statistical methods | 12 | (*a*) Describe all statistical methods, including those used to control for confounding | P10 line170-P11 line 204 |
|  |  | (*b*) Describe any methods used to examine subgroups and interactions | NR |
|  |  | (*c*) Explain how missing data were addressed | NR |
|  |  | (*d*) If applicable, describe analytical methods taking account of sampling strategy | P6 line 98 |
|  |  | (*e*) Describe any sensitivity analyses | P11 line 200-  P12 line 204 |
| Results | | | |
| Participants | 13* | (a) Report numbers of individuals at each stage of study—eg numbers potentially eligible, examined for eligibility, confirmed eligible, included in the study, completing follow-up, and analysed | P12 lines 213-221 |
|  |  | (b) Give reasons for non-participation at each stage | P12 lines 214-215 |
|  |  | (c) Consider use of a flow diagram | NR |
| Descriptive data | 14* | (a) Give characteristics of study participants (eg demographic, clinical, social) and information on exposures and potential confounders | P12 lines 213-221 |
|  |  | (b) Indicate number of participants with missing data for each variable of interest | P12 lines 214-215 |
| Outcome data | 15* | Report numbers of outcome events or summary measures | P14 line 227-P20 line 274 |
| Main results | 16 | (*a*) Give unadjusted estimates and, if applicable, confounder-adjusted estimates and their precision (eg, 95% confidence interval). Make clear which confounders were adjusted for and why they were included | NR |
|  |  | (*b*) Report category boundaries when continuous variables were categorized | NR |
|  |  | (*c*) If relevant, consider translating estimates of relative risk into absolute risk for a meaningful time period | NR |
| Other analyses | 17 | Report other analyses done—eg analyses of subgroups and interactions, and sensitivity analyses | P20 line 275-  P21 line 287 |
| Discussion | | | |
| Key results | 18 | Summarise key results with reference to study objectives | P21 lines 289-303 |
| Limitations | 19 | Discuss limitations of the study, taking into account sources of potential bias or imprecision. Discuss both direction and magnitude of any potential bias | P26 line 386-P27 line 404 |
| Interpretation | 20 | Give a cautious overall interpretation of results considering objectives, limitations, multiplicity of analyses, results from similar studies, and other relevant evidence | P22 line 304-P26 line 385 |
| Generalisability | 21 | Discuss the generalisability (external validity) of the study results | P27 line 405-P28 line 421 |
| Other information | | | |
| Funding | 22 | Give the source of funding and the role of the funders for the present study and, if applicable, for the original study on which the present article is based | Cover letter |

*Give information separately for exposed and unexposed groups.

**Note:** An Explanation and Elaboration article discusses each checklist item and gives methodological background and published examples of transparent reporting. The STROBE checklist is best used in conjunction with this article (freely available on the Web sites of PLoS Medicine at http://www.plosmedicine.org/, Annals of Internal Medicine at http://www.annals.org/, and Epidemiology at http://www.epidem.com/). Information on the STROBE Initiative is available at www.strobe-statement.org.
